# Wild Olive Genotypes as a Valuable Source of Resistance to Defoliating *Verticillium dahliae*

**DOI:** 10.3389/fpls.2021.662060

**Published:** 2021-07-01

**Authors:** Pablo Díaz-Rueda, Ana Aguado, Laura Romero-Cuadrado, Nieves Capote, José M. Colmenero-Flores

**Affiliations:** ^1^Instituto de Recursos Naturales y Agrobiología, Spanish National Research Council (CSIC), Seville, Spain; ^2^Andalusian Institute of Agricultural and Fisheries Research and Training (IFAPA) Centro Las Torres, Seville, Spain

**Keywords:** SILVOLIVE, Verticillium wilt, resistance, *Olea europaea* subspecies, tolerance, olive crop, qPCR

## Abstract

Resistance to the defoliating pathotype of *Verticillium dahliae* has been evaluated in a pool of 68 wild genotypes of olive belonging to the SILVOLIVE collection. Resistance was evaluated by assessing symptom severity using a 0–4 rating scale, estimating the relative area under the disease progress curve (RAUDPC), determining the percentage of dead plants (PDP), and measuring the evolution of morphological parameters in inoculated plants over time. In addition, the density levels of *V. dahliae* in the stem of root-inoculated genotypes have been quantified by means of quantitative real-time PCR at 35 and 120 days after inoculation (dai). Fifteen genotypes (22%) were cataloged as resistant to *V. dahliae* (i.e., disease parameters did not significantly differ from those of the resistant cultivar Frantoio, or were even lower). Resistant genotypes are characterized by presenting fewer symptoms and a lower amount of *V. dahliae* DNA at 120 dai than at 35 dai, indicating their ability to control the disease and reduce the density of the pathogen. The rest of the evaluated genotypes showed variable levels of susceptibility. Overall analysis of all genotypes showed high correlation between symptomatology and the amount of *V. dahliae* DNA in the stem of inoculated genotypes at 120 dai, rather than at 35 dai. However, correlation at 120 dai was not observed in the set of resistant genotypes, suggesting that resistance to defoliating *V. dahliae* in olive is based on the occurrence of different mechanisms such as avoidance or tolerance. These mechanisms are valuable for designing breeding programs and for the identification of target genes and resistant rootstocks to better control Verticillium wilt in the olive grove.

## Introduction

Olive (*Olea europaea* L) is one of the most cultivated woody crops in the world, having high socio-economic importance in all olive-producing areas (Blázquez-Martínez, 1996; Civantos, 2004). Verticillium wilt is considered the most important soilborne disease affecting olive (Bubici and Cirulli, 2011; López-Escudero and Mercado-Blanco, 2011; Tsror, 2011), causing severe losses to growers, nurseries and olive industries (Jiménez-Díaz et al., 2012). The causal agent, *Verticillium dahliae* Kleb., is a soilborne fungus that penetrates the roots of the host plant and colonizes its vascular system. The combined action of the fungal colonization and the defense response of the infected plant, e.g., formation of gels, gums, and tyloses (Fradin and Thomma, 2006; Yadeta and Tomma, 2013) provokes the obstruction of xylem vessels, reducing the transport of water and nutrients and producing wilt symptoms, defoliation, dieback of shoots, and the eventual death of the whole plant (Gharbi et al., 2016). In addition, *V. dahliae* produces mycotoxins that can seriously damage the metabolism of the plant and contribute to plant wilting (Fradin and Thomma, 2006; Luo et al., 2014). Resistance has been evaluated on the basis of external symptoms, vascular browning, and isolation of the fungus from plant tissues (López-Escudero et al., 2004; Martos-Moreno et al., 2006; Colella et al., 2008; Markakis et al., 2009; Bubici and Cirulli, 2012; García-Ruiz et al., 2014) even in naturally infected soils (Trapero et al., 2013). In addition, the qPCR quantification of *V. dahliae* in inoculated plants has been used to determine the resistance of different olive genotypes (Mercado-Blanco et al., 2003; Markakis et al., 2009; Jiménez-Fernández et al., 2016).

*Verticillium dahliae* isolates from olive can be classified into defoliating (D) and non-defoliating (ND) pathotypes, depending on the virulence and the symptoms caused in the infected plant (Schnathorst and Sibbett, 1971; Bejarano-Alcázar et al., 1996). The D pathotype is highly virulent and is able to cause the drop of leaves and the complete death of the tree (Sánchez-Hernández et al., 1998). The ND pathotype is mildly virulent and does not cause defoliation of the tree. Although ND isolates can eventually cause wilting and death of susceptible cultivars, remission of symptoms with time has been observed (López-Escudero and Mercado-Blanco, 2011). The D pathotype has progressively spread and displaced the ND pathotype in most olive cropping areas in southern Spain, severely affecting olive production yields (López-Escudero et al., 2010; Jiménez-Díaz et al., 2011, 2012; Areal and Riesgo, 2014).

Besides having a wide range of host species, including many common crops such as sunflower and cotton (Hiemstra and Harris, 1998; Pegg and Brady, 2002), *V. dahliae* can also asymptomatically colonize other plant species and persist in organic plant residues (Bhat and Subbarao, 1999; Pegg and Brady, 2002). In addition, the fungus is well adapted to long-term survival in soil because it can form resistant structures named microsclerotia (Wilhelm, 1955). All these properties increase the inoculum density in the soil and the dispersion of the pathogen, making the use of crop rotation an unwise strategy to control Verticillium wilt. In recent years, changes in cropping practices including drip irrigation and high-density plantings have also influenced the worsening of the phytosanitary status of olive crops (López-Escudero and Blanco-López, 2005; Villalobos et al., 2006; Pérez-Rodríguez et al., 2015). All these factors, joined to the inefficacy of chemical control, make eradication of *V. dahliae* a very difficult task in olive plantations (López-Escudero et al., 2004; Hegazi et al., 2012; Trapero et al., 2013; García-Ruiz et al., 2014). Because single control measures are generally ineffective, integrated approaches are frequently implemented (López-Escudero and Mercado-Blanco, 2011; Montes-Osuna and Mercado-Blanco, 2020). Cultural methods e.g., control of irrigation, avoiding rotation with susceptible crops, and pruning of branches before defoliation, can contribute to the decrease of the density of inoculum in the soil. Soil solarization, the application of organic amendments to the soil (Varo-Suárez et al., 2018), and the use of biological control agents are sustainable and promising control strategies (Areal and Riesgo, 2014; Gómez-Lama Cabanas et al., 2018; Azabou et al., 2020; Boutaj et al., 2020; Castro et al., 2020; Mulero-Aparicio et al., 2020). Preventive or curative chemical fungicides can only partially control the disease (Tjamos, 1993; Fradin and Thomma, 2006; Tsror, 2011; Gómez-Gálvez et al., 2020). Biotechnological approaches such as the preventive detection of *V. dahliae* by using specific and highly sensitive molecular methods such as quantitative real-time PCR (qPCR), allows the identification of both healthy plant material and free-pathogen pre-planting soils.

The use of resistant cultivars is probably the most effective and sustainable strategy for the control of Verticillium wilt disease. However, most of the commercial cultivars used in Spain (including the most used cultivars Picual, Hojiblanca, Cornicabra, and Arbequina) are susceptible or highly susceptible to *V. dahliae* (López-Escudero et al., 2004; Hegazi et al., 2012; Trapero et al., 2013, 2015; García-Ruiz et al., 2014). The few available resistant cultivars such as Frantoio are not used in olive production because they have lower agronomic quality. However, their use as rootstock has demonstrated to confer resistance to *V. dahliae* to the grafted variety (Porras-Soriano et al., 2003; Bubici and Cirulli, 2012). However, this resistance is slightly broken in soils with moderate and high inoculum density of *V. dahliae* (Trapero et al., 2013) or is not durable in the long term in highly infected soils (Valverde et al., 2021). Therefore, new genotypes with long-lasting resistance to *V. dahliae* are needed as cultivars or rootstocks for adequate control of Verticillium wilt in olive plantations. Breeding programs have been developed with the aim of identifying resistant cultivars (Arias-Calderón et al., 2015a,b,c; Serrano et al., 2021) although satisfactory planting material with appropriate agronomic traits and long-term resistance are not available yet. Several breeding cycles could be necessary to obtain new cultivars with improved agronomic performance. Finally, the use of resistant cultivars (Porras-Soriano et al., 2003; Bubici and Cirulli, 2012) or wild-olive genotypes (Arias-Calderón et al., 2015c; Jiménez-Fernández et al., 2016) as rootstocks has emerged as a feasible alternative for getting *V. dahliae* resistance, although a limited number of genotypes have been evaluated.

The SILVOLIVE collection comprises an extensive number of wild genotypes belonging to all the known subspecies of *Olea europaea*, providing a natural source of genetic variability with high potential to be used for breeding and as rootstocks of commercial varieties (Díaz-Rueda et al., 2020). When used as rootstocks, different genotypes have demonstrated to regulate morphological parameters of the grafted scion and could potentially provide the grafted cultivar with resistance to *V. dahliae.* Taking advantage of the genetic and phenotypic variability of the SILVOLIVE collection, our objectives were (i) to evaluate the resistance to *V. dahliae* of 68 artificially inoculated wild olive genotypes and three control reference cultivars, the highly susceptible Picual, the moderately susceptible Arbequina and the resistant Frantoio; (ii) to determine by qPCR technology if quantification of fungal DNA in the basal stem of inoculated plants is a reliable tool to predict resistance to *V. dahliae* and what is the optimal infection time to assess the degree of susceptibility according to DNA quantification (35 or 120 dai); and (iii) to go in depth into the mechanisms of resistance/tolerance based in the quantification of both fungal density and the development of symptoms at different times after inoculation.

## Materials and Methods

### Plant Material

Plant material consisted in 6 months-old, 20–30 cm high olive plantlets belonging to the SILVOLIVE collection, which comprises genotypes from all known subspecies of *Olea europaea* (*europaea*, *laperrinei, cuspidata, cerasiformis*, *guanchica*, and *maroccana*) (Díaz-Rueda et al., 2020). A total of 71 genotypes were evaluated including 68 wild-olive genotypes, and three commercial varieties (Frantoio, Arbequina, and Picual) used as highly resistant, moderately susceptible, and extremely susceptible reference controls, respectively (Table 1). Wild-olive genotypes were micropropagated from the *in-vitro* SILVOLIVE germplasm collection (Díaz-Rueda et al., 2020). Seedlings were cut into uninodal segments and incubated in Rugini medium (Rugini, 1984) supplemented with 1 mg/L zeatin in a growth chamber with 16 h light photoperiod (34 μM intensity with 70% red: 30% blue light-emitting diodes, LEDs) at 25 ± 2°C. For whole plant regeneration, grown shoots were transferred to rooting medium (50% strength Rugini medium) supplemented with α-naphthalacetic acid (0.8 mg/L). Rooted seedlings were acclimatized *ex-vitro* for 3 weeks, transplanted into 1-L pots and then grown under common greenhouse conditions (Díaz-Rueda et al., 2020).

**TABLE 1:** Mean disease parameters assessed in the wild olive genotypes inoculated with the defoliating isolate VD117 of *Verticillium dahliae*.

**Variety^a^**	**RAUDPC^b^**	**FMS^c^**	**PDP^d^**	**R.L.^e^**
GUA4	83,0	4,0	100	ES
**PICUAL**	**78,0**	**4,0**	**100**	**ES**
FRA1	76,9	4,0	100	ES
AMK25	74,7	3,9	91,7	ES
AJA4	73,3	4,0	100	ES
CUS3	69,1	3,8	92,9	ES
MAR1	69,0	4,0	100	ES
CER3	68,8	4,0	100	ES
GUA1	68,7	4,0	100	ES
GUA2	68,3	3,8	87,5	ES
CUS6	64,0	3,9	87,5	ES
AMK16	63,9	4,0	100	ES
DHO8A	57,4	4,0	100	ES
AJA17	56,7	4,0	100	ES
ACZ10	56,2	3,3	61,5	ES
DHO12A	54,0	3,9	33,3	ES
AMK34	52,6	3,4	0	ES
GUA5	52,4	4,0	93,8	ES
AMK6	51,5	3,3	66,7	ES
AOU4	50,4	4,0	100	ES
AOU11	48,8	3,5	9,1	ES
CUS14	47,5	3,6	88,9	ES
AMS19	44,6	3,3	75	ES
FRA2	44,1	3,9	50	ES
ACZ8	43,4	3,5	38,5	ES
DHO10B	40,1	3,4	80	ES
DHO10A	39,7	3,5	75	ES
AMK9	33,9	2,6	60	ES
DHO6C	33,7	2,8	75	ES
CEH20	21,7	3,9	87,5	ES
GUA8	16,6	3,4	56,3	ES
GUA7	38,7	2,8	16,7	S
AOU10	36,3	2,9	50	S
CER1	36,3	2,9	41,7	S
AMK12	35,4	2,8	50	S
AJA1	34,2	2,8	16,7	S
GUA9	33,3	2,8	43,8	S
FRA3	33,0	2,8	18,2	S
ACZ9	32,5	2,7	12,5	S
CEH21	31,2	3,0	25	S
AMS15	28,7	2,6	14,3	S
TAM4	23,2	3,0	8,3	S
ACZ5	18,6	2,7	25	S
ACZ1	29,2	2,2	12	MS
AMK21	28,9	1,9	33,3	MS
**ARBEQUINA**	**24,2**	**1,9**	**23,1**	**MS**
ARC1	19,3	1,6	13,3	MS
GUA6	16,7	2,0	11,1	MS
ACZ7	16,4	1,9	0	MS
AJA12	15,7	1,0	16,7	MS
ACO18	14,5	1,3	20	MS
TAM3	10,0	1,5	33,3	MS
DHO1	9,3	1,4	16,7	MS
AJA6	9,3	1,8	0	MS
APR1	5,0	1,6	0	MS
AMK27	10,8	0,9	0	R
AMK14	9,6	0,9	0	R
CEH23	9,1	1,0	0	R
AMS17	5,1	0,7	0	R
CEH8	4,3	0,7	0	R
AOU3	4,3	0,6	0	R
**FRANTOIO**	**2,7**	**0,5**	**0**	**R**
DHO6A	2,2	0,3	0	R
ACO1	2,0	0,6	0	R
ACO14	1,9	0,3	0	R
DHO6B	1,6	0,4	0	R
AMK5	1,3	0,1	0	R
ACZ3	0,6	0,3	0	R
TAM12	0,4	0,1	0	R
ACO15	0,4	0,1	0	R
GUA3	0,2	0,0	0	R

*^*a*^Genotypes from the SILVOLIVE collection (Díaz-Rueda et al., 2020). ^*b*^RAUDPC: Relative area under the disease progress curve estimated as the percentage with regard to the potential maximum value. ^*c*^FMS, final mean severity of symptoms. ^*d*^PDP, percentage of dead plants. ^*e*^R.L., resistance level of each genotype (López-Escudero et al., 2007). ES, extremely susceptible; S, susceptible; MS, moderately susceptible; R, resistant. Reference control cultivars “Picual” (extremely susceptible), “Arbequina” (moderately susceptible), and “Frantoio” (resistant) are indicated in bold.*

### Fungal Isolate and Inoculum Production

The defoliating *V. dahliae* pathotype VD-117, obtained from the culture collection of IFAPA research center, Córdoba (Spain) (Bejarano-Alcázar et al., 1996), was used in the inoculation tests for resistance assessment. Conidial suspensions for resistance tests were prepared by transferring five 8 mm-Potato Dextrose Agar (PDA) discs of actively growing mycelium of VD-117 isolate to flasks containing 100 mL potato dextrose broth (PDB) and incubated at 150 rpm in an orbital shaker at 24°C in the dark for 7 days. The conidial suspension was filtered through four layers of sterile cheesecloth and adjusted to 1 × 10^7^ conidia/ml with sterile distilled water using a haemocytometer.

### Inoculation of Olive Plants

For assessing resistance to *V. dahliae* in the 68 wild-olive genotypes (Supplementary Figure 2), four experiments were carried out over four different years: 2016, 2017, 2018, and 2019. The reference cultivars were repeated in the four trials, and a number of wild genotypes have been repeated in, at least, two different assays. Thus, ACZ1 and ACO15 have been repeated in three different assays while APR, ACZ7, ACZ9, ARC, GUA8, GUA9, CUS6, GUA5, AMK16, AMK5, AMK6, and DHO6A have been repeated in two different assays. Repetitions of both reference cultivars and wild-type genotypes in different trials and years have shown consistent results. Twenty-four plants were inoculated for each genotype. Plant roots were washed under abundant tap water to remove the substrate. Secondary roots were cut in 4–5 positions and the whole bare root system was dipped in the *V. dahliae* conidial suspension for 15 min. Control plants were treated by immersion of roots in PDB:sterile distilled water (1:1, v:v). Each plant was individually transplanted into 1 L pots containing sterilized 2:1 silt:peat moss (v:v). A completely randomized blocks design with four blocks and six plants (repetitions) per block for each genotype was used. Plants were maintained at 24/18°C and 60/40% relative humidity (day/night) in a greenhouse with a daily 14-h photoperiod supplemented with fluorescent illumination (360 mmol/m^2^). Plants were watered as needed, and fertilized weekly with Hoagland’s nutrient solution (Hoagland and Arnon, 1950). The most resistant genotypes from each pathogenicity test were re-evaluated in the following experiment, so that susceptible and highly susceptible genotypes were evaluated once, whereas moderately susceptible and resistant genotypes were evaluated twice.

### Symptoms Assessment

Symptoms of aerial organs were evaluated on each plant every 2 weeks, starting 35 days after inoculation. Symptoms were registered following a 0–4 rating scale according to the percentage of Maximum Intensity Symptoms (*MIS*): chlorosis, leaf curl, stunting, leaf and shoot necrosis or defoliation: 0 = 0% *MIS* or no symptoms; 1 = 25% *MIS*; 2 = 50% *MIS*; 3 = 75% *MIS*; 4 = 100% *MIS* or dead plants.

At the end of the experiment, the following disease parameters were estimated from these scale values: (i) the relative area under the disease progress curve (RAUDPC), calculated for each cultivar considering its percentage with regard to the maximum possible value that could be reached in the period of assessment, based on the calculation formula according to (Campbell and Madden, 1990):

(1) $$R\,A\,U\,D\,P\,C\,\left[\sum_{i\,1}^{n}\left(\frac{S_{i}\,S_{i-1}}{2}\right)\,△\,t\right]\,\left[\frac{100}{S_{m\,a\,x}\,T}\right]$$

where S_*i*_ = mean severity of the experimental unit in the observation i; △*t* = the number of days between observations; S_max_ = maximum disease rating (=4); T = experimental period in days (=120); *n* = number of observations; (ii) the final mean severity of symptoms (FMS), calculated according to López-Escudero et al. (2007); (iii) the percentage of dead plants (PDP) from the total of inoculated plants. Using all these parameters, the resistance level (RL) of each genotype was determined according to (López-Escudero et al., 2007): ES = extremely susceptible; S = susceptible; MS = moderately susceptible; R = resistant. In assay 3, morphological parameters such as the number of nodes, the accumulated length of branches, and the plant height of each plant were also measured over time at 0, 35, 50, 70, 85, 100, and 120 dai. The relative growth rate (RGR) was calculated as the number of new nodes per day.

### Quantification of *Verticillium dahliae* in Plant Tissues by Real-Time PCR

Quantification of *V. dahliae* levels in the inoculated plants was carried out by qPCR at two times, 35 and 120 dai, using half of the inoculated plants for each time of analysis (three inoculated plants/block and four blocks, in a total of 12 plants per analyzed time). Plants were removed from the pots and two thirds of the stem, corresponding to the basal portion, was deprived of leaves, and immediately frozen at −80°C until analysis. Stems of plants from the same experimental block were grouped in a composite sample, giving rise to four biological replicates per treatment and harvesting time. Samples were ground in a mortar in presence of liquid nitrogen until getting a fine powder. Total DNA was extracted using the Isolate II Plant DNA Kit (Bioline, London, United Kingdom) following the manufacturers’ instructions. DNA concentration was accurately determined in triplicate measurements by using a fluorescent spectrophotometer (Modulus^TM^ II Microplate Multimode reader, Turner Biosystems, United States). PCR reactions amplifying an intergenic spacer of *V. dahliae* previously characterized (Bilodeau et al., 2012) were performed in a 20 mL final volume, in 96-well plates in a CFX Connect thermocycler (Bio-Rad). The reaction cocktail contained 1x SensiMix (SensiMix^TM^ Probe Kit, Bioline), 1 mM each forward and reverse primers (Bilodeau et al., 2012), 5 mM TaqMan probe labeled with 6′FAM fluorescein (Bilodeau et al., 2012), 0.1 mg/mL BSA, and 3 μL of extracted DNA adjusted to a concentration of 15 ng/mL. Amplifications were carried out at 95°C for 10 min, and 55 cycles of 15 s at 95°C and 30 s at 62°C. For normalization of DNA loading, a conserved region of the plant housekeeping cytochrome oxidase gene (*cox*) was amplified in a parallel qPCR reaction with the same conditions as those used for *V. dahliae* amplification (Garrido et al., 2009). The standard curve for *V. dahliae* quantification was obtained through serial dilutions of genomic DNA from *V. dahliae* VD-117 (10 ng to 1 fg) in a fixed background of plant DNA (20 ng/ml) obtained from healthy olive stems. *V. dahliae* DNA was extracted from 0.1 g VD-117 mycelium grown for 10-days in PDA medium, using the Isolate II Plant DNA Kit, as described above. The standard curve for *cox* quantification was obtained through serial dilutions of DNA from non-treated olive plants (10 ng to 0.1 pg). The relative amounts of *V. dahliae* and *cox* DNA in the inoculated olive plants were obtained by extrapolation of cycle threshold value from the respective standard curves through the CFX Manager software (Bio-Rad). The threshold position of the DNA standard curves generated from different plates was manually fixed at the same position for all treatments and experiments for better comparison (Vaerman et al., 2004). The efficiencies of the reactions were calculated from the slope of the standard curves according to the following formula: *E* = 10 ^(–1/slope)^. Four biological replicates (one DNA extractions × each block × each time) and three technical replicates (PCR reaction) of standards, samples and DNA template-free controls were used. Results were expressed as means ± standard errors of the *V. dahliae*:*cox* ratios (ng *V. dahliae* DNA ⋅ ng plant DNA^–1^), previously defined as mean normalized quantity (MNQ) in Garrido et al. (2009). Quantifications were also calculated as ng of *V. dahliae* DNA per ng of plant DNA, and similar quantitative results were obtained than when using *cox* normalization (data not shown).

### Statistical Analysis and Normalization

Statistical analyses were performed with Statistix 9.0 (Analytical Software, Tallahassee, FL, United States). Data from varieties that were repeated twice were subjected to analysis of variance (ANOVA) using the assays as a factor. Where significant differences were not detected, data were pooled for the calculation of the means. For those varieties repeated with differences in their results, the chosen data was that with the highest severity. Means of FMS, RAUDPC and DNA content at 120 dai were compared between each genotype and the means of Frantoio, Arbequina, and Picual reference controls, respectively, by the LSD test at *P* = 0.05. Percentage data were previously transformed by arcsine (Y/100)^1/2^. Linear regression analyses were performed for estimating relationships between biometric parameters and FMS or RAUDPC in inoculated plants at 120 dai, respectively.

## Results

### Development of Symptoms in Inoculated Wild Olive Genotypes

**FIGURE 1:**
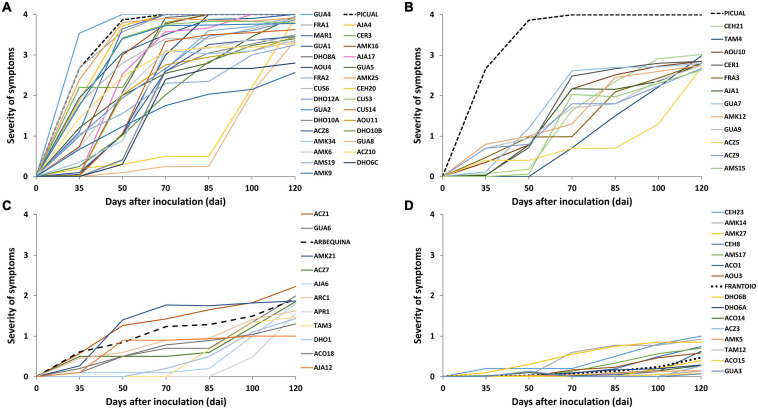
Progress of the severity of symptoms recorded in wild olive genotypes inoculated with the defoliating isolate VD117 of *Verticillium dahliae* (assays 1–4). Values are the means of 24 plants per assay. Severity of symptoms was assessed each time on a 0–4 rating scale according to the percentage of Maximum Intensity Symptoms (*MIS*): chlorosis, leaf and shoot necrosis or defoliation: 0 = 0% *MIS* or no symptoms; 1 = 25% *MIS*; 2 = 50% *MIS*; 3 = 75% *MIS*; 4 = 100% *MIS* or dead plants. **(A)** extremely susceptible, **(B)** susceptible, **(C)** moderately susceptible, and **(D)** resistant genotypes. The extremely susceptible cultivar Picual was maintained in panel 1B as a reference. Reference control cultivars Picual, Arbequina, and Frantoio are indicated in dashed lines.

**FIGURE 2:**
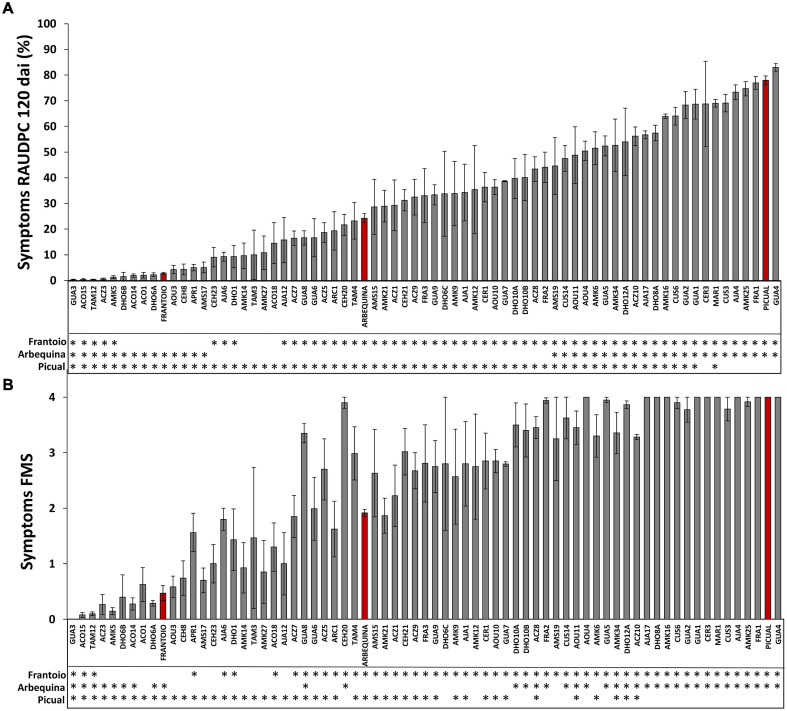
Symptoms of infection of olive genotypes inoculated with the *Verticillium dahliae* defoliating isolate VD117. The Relative Area Under the Disease Progress Curve (RAUDPC) **(A)** and the Final Mean Severity (FMS) **(B)** of all wild olive genotypes was evaluated at 120 days after inoculation (dai). Red bars correspond to the reference cultivars: the resistant Frantoio (RAUDPC < 10, FMS < 1.5 and PDP = 0); the moderately susceptible Arbequina (RAUDPC 31–50, FMS 1.5–2.5 and PDP = 0–30); and the extremely susceptible Picual (RAUDPC > 71, FMS > 3.0 and PDP > 51), according to the levels of resistance described by López-Escudero et al. (2007). Asterisks indicate significant differences with cvs. Frantoio, Arbequina, and Picual, respectively. Results are the mean of 24 plants and error bars correspond to the standard error. Data were subjected to analysis of variance (ANOVA) and multiple comparisons of means were analyzed by LSD (Least Significant Difference) at *P* = 0.05. Multiple range test was performed by Statistix 9.0 (Analytical Software, Tallahase, FL, United States).

In preliminary assays, not reported here, quantification of *Verticillium* DNA content and symptoms was carried out at times shorter than 120 dai. We noticed that, in order to obtain clear results on the resistance/tolerance mechanisms displayed by olive wild genotypes, both *Verticillium* DNA content and symptoms had to be recorder in 120 dai trials. The inoculated cultivars used as control references developed symptoms of Verticillium wilt according to their previously established RLs (Table 1, Figure 1, and Supplementary Figure 1). Thus, Picual plants presented severe wilting symptoms, showing early defoliation around 21 dai, chlorosis, leaf curl, leaf and shoot necrosis, stunting, and death. Picual plants exhibited a RAUDPC of 78.0% and reached a final mean severity (FMS) index of 4 on the 0–4 symptoms scale. Arbequina, considered as moderately susceptible, showed mild symptoms starting around 50 dai, consisting on chlorosis, moderate defoliation, leaf, and shoot necrosis. RAUDPC and FMS values in Arbequina plants were 24.2%, and 1.9, respectively. Frantoio plants behaved as resistant and displayed very slight symptoms showing occasional defoliation or leaf curl, although most of the inoculated plants did not show symptoms. Frantoio plants achieved a RAUDPC value of 2.7%, and an FMS value of 0.5. At the end of the experiment, the PDP was 100% for Picual, 23.1% for Arbequina and 0% for Frantoio varieties. RAUDPC, FMS, and PDP values were significantly different (*P* < 0.05) between each of the Picual, Arbequina, and Frantoio cultivars (Figure 2).

A wide variability of the disease progress was observed in wild olive genotypes (Supplementary Figure 1). Wild olive genotypes and reference olive cultivars were classified into four groups of resistance (Table 1) according to the criteria defined by López-Escudero et al. (2007): extremely susceptible (Figure 1A); susceptible (Figure 1B); moderately susceptible (Figure 1C); and resistant (Figure 1D) genotypes. Thirty out of 68 wild olive genotypes assayed (44%) were extremely susceptible to the defoliating isolate VD117 of *V. dahliae*. With the exception of DHO12A, AMK34, AOU11, and ACZ8, all extremely susceptible genotypes showed a mortality rate of at least 50% at 120 dai (Table 1). A group of 12 genotypes (18%) exhibited RAUDPC values ranging from 18.6 to 38.7%, FMS between 2.6 and 3.0, and PDP from 8.3 to 50%, and were considered as susceptible (Table 1 and Figure 1B). Eleven genotypes (16%) showed disease parameters similar to those of Arbequina, i.e., RAUDPC values from 5.0 to 29.2%, FMS between 1.0 and 2.2, and PDP from 0 to 33.3%, and were classified as moderately susceptible (Table 1 and Figure 1C).

A group of 15 wild olive genotypes (22%) were defined as resistant and presented low RAUDPC values (0.2–10.8%), moderate or no symptoms (FMS from 0.0 to 1.0), and no dead plants (PDP 0%) (Table 1 and Figure 1D). Within this group, genotypes GUA3, ACO15, TAM12, ACZ3, and AMK5 exhibited lower symptoms than Frantoio, with significantly lower values in RAUDPC and/or FMS parameters, pointing to greater resistance to Verticillium wilt than the resistant cultivar of reference (Figure 2). The resistant genotypes DHO6B, ACO14, ACO1, DHO6A, AOU3, CEH8, AMS17, AMK14, and AMK27 showed non-significant differences with the resistant cultivar Frantoio in RAUDPC and FMS values (Figure 2).

**FIGURE 3:**
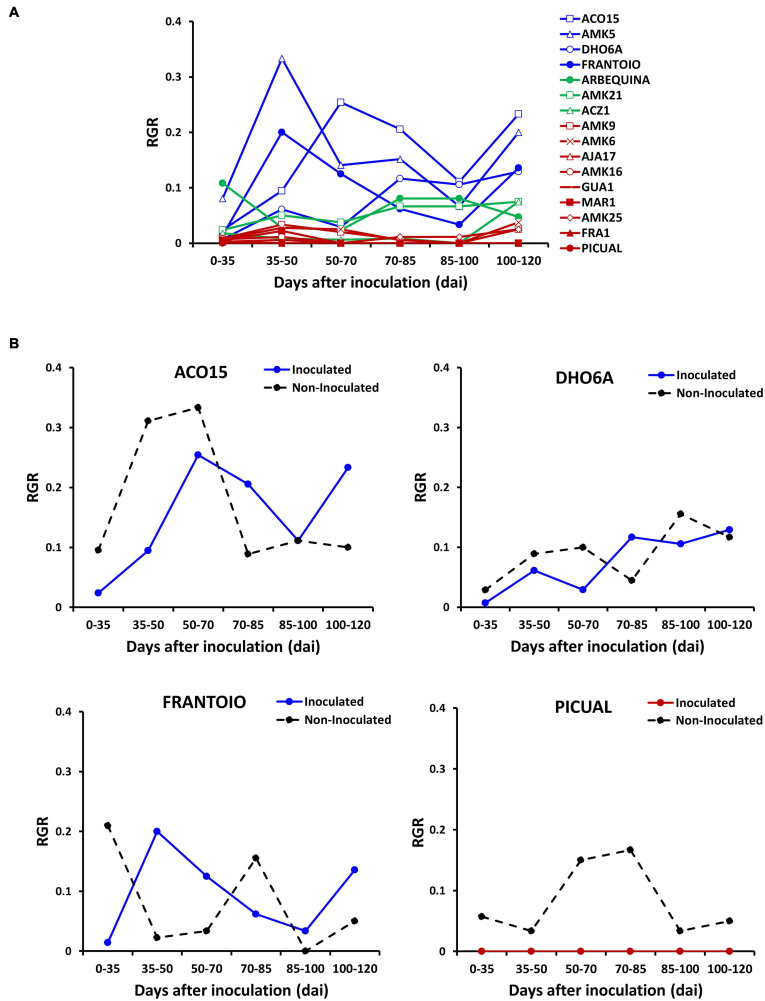
Relative growth rate (RGR) quantified as the production of new nodes per day in wild genotypes and cultivars of assay 3. **(A)** RGR values of plants inoculated with the defoliating isolate VD117 of *Verticillium dahliae* were compared between resistant (blue), moderately susceptible (green) and susceptible or extremely susceptible (red) genotypes. **(B)** Comparison of inoculated (plain lines) vs. non-inoculated (dashed black lines) RGR in resistant wild genotypes and in representative cultivars: resistant Frantoio and extremely susceptible Picual.

**FIGURE 4:**
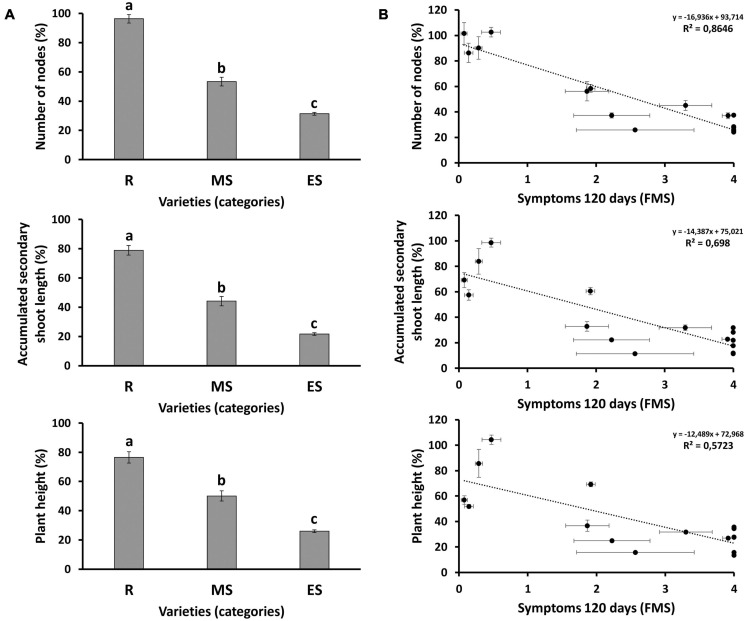
**(A)** Morphological parameters in different groups of susceptibility at 120 days after inoculation (dai). Results are given as the average value of the different genotypes included in each group of susceptibility: R, resistant genotypes; MS, moderately susceptible genotypes; ES, extremely susceptible genotypes. For each genotype, the morphological value was normalized with the average value of the respective non-inoculated plants. Letters above bars represent significant differences at *P* < 0.05. **(B)** Correlations between morphological parameters and symptoms, measured as final mean severity of symptoms (FMS) at 120 dai. Scatter plots including the regression line, the regression equation and the coefficient of determination (*R*^2^) are given for each morphological parameter: number of nodes; accumulated secondary shoot length; and plant height. Bars and dots represent the average value of 12 plants normalized with the average value of the respective non-inoculated plants for each genotype. Error bars correspond to standard errors of the mean.

Evolution of morphological parameters after treatment was measured in all genotypes analyzed in assay 3 (Figure 3 and Supplementary Figure 2). After inoculation, the relative growth rate was strongly inhibited in susceptible, but not in resistant genotypes (Figure 3A). Resistant genotypes inoculated with the fungus showed little or no growth inhibition when compared to their non-inoculated controls (Figure 3B). In contrast, a remarkable growth inhibition was detected in the inoculated Arbequina (Figure 3A), and full growth inhibition was observed in the inoculated Picual susceptible cultivar (Figure 3B). Production of new nodes was similar in inoculated and non-inoculated resistant genotypes compared to the scarce or null production of new nodes observed in susceptible genotypes (Supplementary Figures 2A,B, respectively). Accumulated secondary shoot length was more inhibited in susceptible than in resistant genotypes (Supplementary Figures 2C,D). This is, at least in part, a result of the reduction of the stem internodal elongation in inoculated plants (Supplementary Figures 2E,F). At the end of the experiment, average growth inhibition by Verticillium wilt (relative to non-inoculated plants), measured as production of new nodes, accumulated secondary shoot length, and plant height, was significantly lower in the group of resistant genotypes than in the moderately or extremely susceptible ones (Figure 4A). Significant negative correlations were observed between FMS and the production of new nodes (*R*^2^ = −0.8646); the accumulated secondary shoot length (*R*^2^ = −0.698); and the plant height (*R*^2^ = −0.5723), respectively (Figure 4B).

### Quantification of *Verticillium dahliae* in Inoculated Wild Olive Genotypes

**FIGURE 5:**
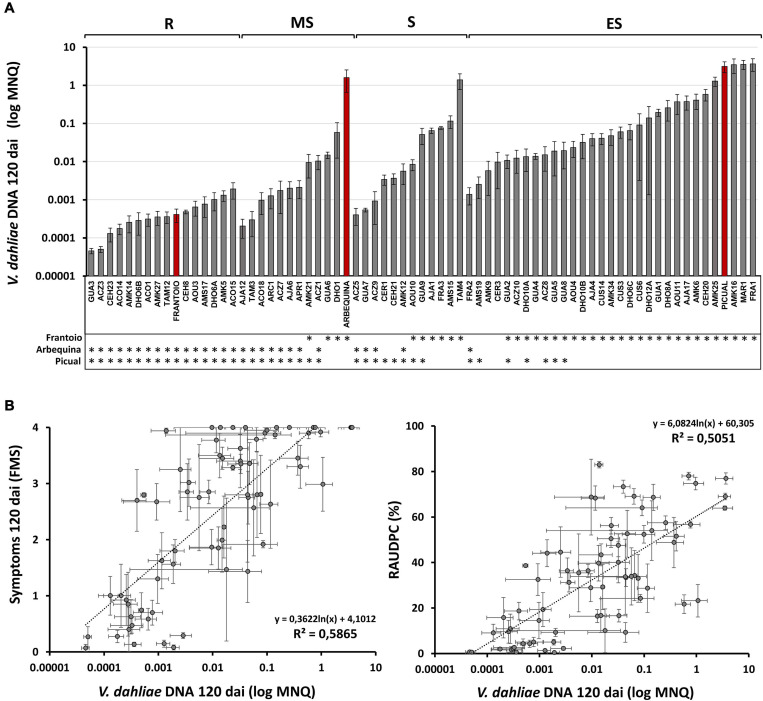
**(A)** Mean Normalized Quantity (MNQ) of *Verticillium dahliae* DNA of wild olive genotypes evaluated at 120 days after inoculation (dai) with the *V. dahliae* defoliating isolate VD117. Red bars represent cvs. Frantoio, Arbequina, and Picual as reference cultivars of resistant, moderately susceptible, and extremely susceptible genotypes, respectively, according to the resistance level described by López-Escudero et al. (2007). Asterisks indicate significant differences with cvs. Frantoio, Arbequina, and Picual, respectively. **(B)** Scatter plots including regression line, regression equation and coefficient of determination (*R*^2^) for the relationships between the Mean Normalized Quantity (MNQ) of *V. dahliae* DNA and the Final Mean Severity (FMS) (left) or the RAUDPC (right) at 120 dai. Results are the mean of 12 plants per assay, or 24 plants for those genotypes repeated in two different assays. Error bars in two dimensions indicate the standard error of the mean.

The limit of detection of *V. dahliae* in the basal stems of inoculated plants using the TaqMan-based qPCR protocol was 15 fg of fungal DNA. No differences between the efficiency and Ct values of the standard curves were found between experiments, so that the data from experiments 1–4 could be compared. Average values of *V. dahliae* DNA content (MNQ) in the basal stem of the reference cultivars at 120 dai were: 3,130.93 × 10^–3^ in the susceptible Picual; 1,594.41 × 10^–3^ in the moderately susceptible Arbequina; and 0.41 × 10^–3^ in the resistant Frantoio (Supplementary Table 1 and Figure 5A).

According to the classification of RLs of genotypes shown in Table 1, average MNQ values of *V. dahliae* DNA at 120 dai ranged as follows (Supplementary Table 1): from 3,658.79 × 10^–3^ (FRA1) to 1.39 × 10^–3^ (FRA2) in extremely susceptible genotypes; from 1,392.34 × 10^–3^ (TAM4) to 0.40 × 10^–3^ (ACZ5) in susceptible genotypes; from 58.25 × 10^–3^ (DHO1) to 0.2 × 10^–3^ (AJA12) in moderately susceptible genotypes; and from 1.90 × 10^–3^ (ACO15) to 0.04 × 10^–3^ (GUA3) in resistant genotypes (Supplementary Table 1). Therefore, the highest amounts of *V. dahliae* DNA were detected in extremely susceptible genotypes, whereas the lowest DNA amounts of *V. dahliae* DNA were detected in resistant genotypes. In the group of resistant genotypes, GUA3, ACZ3, CEH23, ACO14, AMK14, DHO6B, ACO1, AMK27, and TAM12 showed lower content of *V. dahliae* DNA than the resistant cultivar Frantoio at 120 dai, although differences were not statistically significant. The lowest content of *V. dahliae* DNA was detected in ACZ3 and GUA3 genotypes, about ten times lower than that of the resistant Frantoio.

### Correlation Patterns Between *Verticillium dahliae* DNA Content and Plant Symptoms

In agreement with the results previously shown, a statistically significant positive linear correlation was established between the amount *V. dahliae* in plant shoot tissues and the plant symptoms at 120 dai, measured as FMS (*R*^2^ = 0.5865) or RAUDPC (*R*^2^ = 0.5051) (Figure 5B). This positive correlation was observed not only when the genotypes of the different assays were analyzed together (Figure 5B), but also when they were analyzed as separated assays (Supplementary Figure 3). However, in the subset of resistant genotypes, no correlation could be observed between the *V. dahliae* DNA content and symptoms at 120 dai calculated as RAUDPC (*R*^2^ = 0.1987) or FMS (*R*^2^ = 0.0202) (Supplementary Figure 4A). DNA of *V. dahliae* was also quantified at 35 dai (in assays 1, 2, and 3), but a poor correlation between the amount of *V. dahliae* DNA and symptoms was observed at this early period of infection (FMS *R*^2^ = 0.21; Supplementary Figure 4B). And no correlation at all was observed between *V. dahliae* DNA content at 35 dai and symptoms at 120 dai (FMS *R*^2^ = 0.0782; Supplementary Figure 4B), suggesting that diagnosis of Verticillium wilt based upon DNA quantification cannot be performed during an early stage of infection.

**FIGURE 6:**
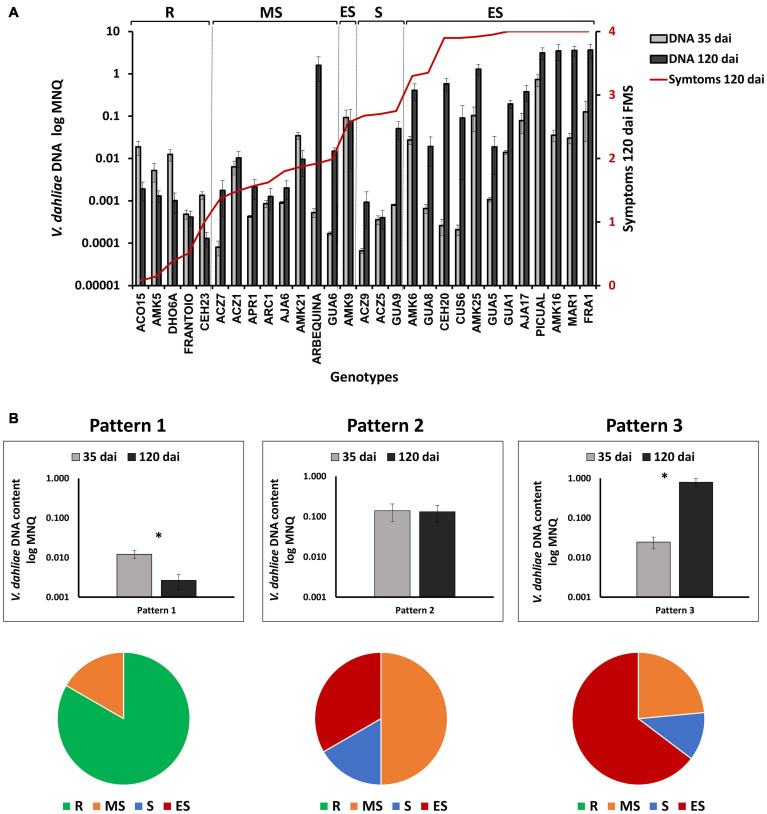
**(A)** Evolution of *Verticillium dahliae* content after inoculation in genotypes tested in assays 1, 2, and 3 **(A)** Quantification of *V. dahliae* DNA (Mean Normalized Quantity, MNQ) in wild olive genotypes inoculated with the defoliating isolate VD117 of *V. dahliae* at 35 and 120 days after inoculation (dai). Bars represent the mean of 12 plants per assay, or 24 plants for those genotypes repeated in two different assays. The red line represents the average final mean severity (FMS) at 120 dai for each genotype. **(B)** Patterns of variation of *V. dahliae* DNA quantity between 35 and 120 dai (upper diagram), and relative content of genotypes according to the resistance level described by López-Escudero et al., 2007; lower diagram) for each pattern. Asterisks indicate significant differences at *P* < 0.05. The data were subjected to analysis of variance (ANOVA) and multiple comparisons of means were analyzed by Tukey’s HSD (Honestly significant difference). Error bars represent the standard error of the mean. R, resistant; MS, moderately susceptible; S, susceptible; ES, extremely susceptible.

We took advantage of having available DNA content values at early and late infection periods in assays 1, 2, and 3, to study the occurrence of different evolution patterns of infection over time, and to compare it with the degree of susceptibility to the disease in the different genotypes (Figure 6A). We observed that the lack of correlation between *V. dahliae* DNA content and symptoms in the group of resistant genotypes was even more pronounced at 35 than at 120 dai (Supplementary Figure 5). Thus, genotypes with very low or no symptoms such as ACO15 and DHO6A, showed significantly higher *V. dahliae* DNA content at 35 dai than genotypes such as Frantoio or CEH23, with lower *V. dahliae* DNA and higher symptoms (Supplementary Figure 5 and Figure 2). The data suggest the occurrence of genotypes such as ACO15 and DHO6A that tolerate the presence of relatively high amounts of the fungus. Therefore, different response patterns in the evolution of the fungus in plant tissues after infection can be distinguished (Figure 6B). The first one (pattern 1) consisted of a significant decrease in the amount of *V. dahliae* DNA between 35 and 120 dai. All the resistant genotypes, and the moderately susceptible AMK21 genotype, followed this pattern (Figure 6B). Pattern 2 showed no significant variation in the quantity of *V. dahliae* DNA between 35 and 120 dai. This pattern included 43% of moderately susceptible, 33% of susceptible, and 15% of extremely susceptible genotypes to *V. dahliae*, but none of the resistant genotypes (Figure 6B). Finally, in pattern 3, a significant increase in *V. dahliae* DNA at 120 dai respect to 35 dai was observed, including most of extremely susceptible (85%), 67% susceptible, 43% moderately susceptible genotypes, and none of the resistant genotypes (Figure 6B). The results point to the occurrence of different mechanisms of resistance to Verticillium wilt in wild olive genotypes.

## Discussion

Control of Verticillium wilt in olive is nowadays a challenge that must be addressed under an integrated management strategy (López-Escudero and Mercado-Blanco, 2011; Montes-Osuna and Mercado-Blanco, 2020), in which the search for sources of resistance should be of highest priority. Most of the commercial olive varieties domesticated for higher fruit and olive oil yields and quality are susceptible to *V. dahliae*. First steps in the search for resistant genotypes based on germplasm collections composed of commercial varieties, wild genotypes or breeding crosses have been addressed (Caballero and Del Río, 2008; Arias-Calderón et al., 2015c; Jiménez-Fernández et al., 2016), but still with no practical transference of results to olive production. In this work, the resistance to a defoliating isolate of *V. dahliae* has been evaluated for a high number of wild olive genotypes belonging to the SILVOLIVE collection. This collection is composed of individuals representative of all known subspecies of *Olea europaea* (*europaea*, *cuspidata, laperrinei*, *cerasiformis*, *guanchica*, and *maroccana*), which confers it a high diversity and genetic variability (Díaz-Rueda et al., 2020). Our results provide keys to better understand the resistance mechanisms to Verticillium wilt and a source of genotypes to be used in breeding programs or as rootstocks to improve the control of the disease in the olive grove.

### Wild Olive Genotypes: A Valuable Source of Resistance to Verticillium Wilt

A wide spectrum of RLs was found among the SILVOLIVE genotypes tested (Table 1 and Figures 1, 2). This is in accordance with the variability of RLs previously described in olive cultivars, wild olive genotypes, and the offspring of breeding crosses (Colella et al., 2008; Arias-Calderón et al., 2015b; Jiménez-Fernández et al., 2016). The percentage of resistant genotypes found in our work was 22% (15 out of 68), similar to another screening of comparable dimensions that reported 23% (13 out of 55) resistant genotypes (Arias-Calderón et al., 2015c). The resistant genotypes displayed similar or even better behavior than the resistant cultivar Frantoio, i.e., delay of the disease progress, scarce wilt symptoms, lower reduction of growth parameters, and no incidence of dead plants (Table 1 and Figures 1–3). The mean content of *V. dahliae* DNA quantified in the stem of root-inoculated resistant genotypes was 1,647 (ACO15) to 68,043 (GUA3) times lower than that of the susceptible cultivar Picual. Other genotypes previously reported as resistant showed DNA contents that were 249–1,537 times lower than Picual (Jiménez-Fernández et al., 2016). The content of *V. dahliae* DNA was 837 (ACO15) to 34,565 (GUA3) times lower than that of the moderately susceptible Arbequina, which is the most widely used cultivar in super-intensive olive orchards. Four of the six subspecies of *O. europaea* are represented in the resistant pool of the SILVOLIVE collection (*europaea*, *cuspidata*, *laperrine*, and *guanchica*) with no relationship between resistance and olive subspecies. We have not found resistant genotypes among the subspecies *maroccana* and *cerasiformis*, probably due to the low number of genotypes assayed (MAR1 from subsp. *maroccana* and CER1 and CER3 from subsp. *cerasiformis*). These genotypes were classified as susceptible (CER3) or extremely susceptible (MAR1, CER1) to *V. dahliae* despite being polyploid genotypes, a characteristic previously related to resistance to abiotic and biotic stress in plants (Sattler et al., 2016; Ruiz et al., 2020; Russo et al., 2020).

The presence of genotypes that showed significant higher RLs than Frantoio is remarkable (Figure 2). The resistant genotype GUA3 belongs to the subspecies *guanchica* (Canary Islands, Spain). Progenies of this subspecies have been previously reported as resistant to the D pathotype of *V. dahliae* (Arias-Calderón et al., 2015b). However, most of the *guanchica* genotypes assayed in our study were classified as susceptible (GUA7 and GUA9) or extremely susceptible (GUA1, GUA2, GUA4, GUA5, GUA7, and GUA8), indicating that *guanchica* subspecies include genotypes with different levels of resistance to the fungus.

*Verticillium dahliae* inoculation did not significantly inhibit the relative growth rate of resistant genotypes (Figure 3). Most susceptible genotypes showed severe reduction of the internodal length (Supplementary Figure 2E), probably as a result of impaired cell elongation. This phenomenon may be due to the loss of hydraulic conductivity in infected plants as a consequence of vascular occlusion by accumulation of defense metabolites such as tyloses and gels (Yadeta et al., 2013; Gharbi et al., 2017) or by cavitation of xylem vessels (Pouzoulet et al., 2014; Trapero et al., 2018).

### Strategies for the Control of Verticillium Wilt in Resistant Genotypes

Different patterns of *V. dahliae* DNA at 35 and 120 dai *vs*. different degree of symptoms developed by wild genotypes, point to the occurrence of different mechanisms of resistance to Verticillium wilt. On the one hand, we propose that genotypes with relatively high content of *V. dahliae* at 35 dai and low level at 120 dai (e.g., ACO15, AMK5, and DHO6A) tolerate moderate infection levels at the short term, and control the infection at the medium- and long-term, reducing the amount of fungus in the plant tissues and exhibiting minimal symptoms (Figures 2, 3). On the other hand, genotypes like Frantoio and CEH23 maintained low *V. dahliae* DNA levels at 35 and 120 dai, suggesting a more effective disease avoidance ability, since they prevent the fungus to proliferate in the shorter and longer terms. The occurrence of the two resistance mechanisms, tolerance and avoidance, may be the reason explaining the lack of correlation between the content of *V. dahliae* DNA and symptoms in the pool of resistant genotypes (Supplementary Figure 5). Anyway, it is clearly stated here the fact that resistant genotypes limit the spread of *V. dahliae*. Thus, a significant decrease in the amount of fungus DNA at 120 days compared to 35 days is observed in most resistant plants, a phenomenon that did not occur in non-resistant genotypes.

Different physiological, cellular and molecular mechanisms of resistance have been proposed in *V. dahliae* resistant genotypes (Gómez-Lama Cabanas et al., 2015; Trapero et al., 2018): the reinforcement of the cell wall by deposition of lignin and suberin at the site of infection (Gharbi et al., 2017); the production of reactive oxygen species such as H_2_O_2_ (Gharbi et al., 2017); and the early activation of plant defense mechanisms (Gharbi et al., 2016), such as the induction of genes coding for chitinases and β-1,3-glucanase to degrade the pathogen cell wall. Elucidating what type of molecular mechanisms determines the tolerance vs. the avoidance response to Verticillium wilt is of prime interest.

### Potential Use of Genotypes as Resistant Rootstocks

Therefore, grafting susceptible cultivars of economic relevance, such as Picual and Arbequina, onto resistant genotypes is a necessary approach to identify the most convenient strategy of resistance. From the resistance mechanisms previously proposed, tolerance to *V. dahliae* would be optimal to be implemented in cultivars through breeding programs, while the disease avoidance would be optimal for rootstocks. Thus, although exhibiting minimal symptoms, the tolerance strategy of ACO15, AMK5, and DHO6A may have the disadvantage of allowing the fungus to proliferate and reach the grafted scion during the early infection period. From this perspective, the strategy of minimizing the proliferation of the fungus in the rootstock, represented by the resistant Frantoio and CEH23 genotypes, could be more appropriate. We are currently conducting these assays with grafted plants to clarify these points.

These and other resistant genotypes can be used as rootstocks to improve Verticillium wilt resistance in the grafted plant as previously shown (Bubici and Cirulli, 2012; Porras-Soriano et al., 2003). Furthermore, other traits previously characterized in these genotypes make them of special interest for their potential use as rootstocks. Thus, ACO15, AMK5, and DHO6A (*europaea* subspecies, Marrakech, Morocco), ACZ3 (*europaea* subspecies, Cádiz Mountains, Spain) and TAM12 (*europaea* subspecies, Tamri, Morocco) were classified as very low to intermediate vigor and high branching genotypes. Vigor reduction is a desirable trait in genotypes to be used as rootstocks for high and super high-density hedgerow orchards, a trait that can be transmitted to the grafted scion (Díaz-Rueda et al., 2020). It may be also the case of high branching, which means increased canopy density and high number of potential fruiting sites.

### DNA Quantification of *Verticillium dahliae* as a Tool for Diagnosis of Verticillium Wilt

*In planta* quantification of *V. dahliae* DNA through TaqMan qPCR technology allowed specific detection and accurate quantification of the pathogen in this work, as previously shown (Mercado-Blanco et al., 2003; Gramaje et al., 2013; Jiménez-Fernández et al., 2016). DNA of *V. dahliae* was detected in all genotypes assayed, including the highly resistant ones, demonstrating that the fungus penetrated the root and colonized the stem of the plant. The lack of correlation between *V. dahliae* DNA at 35 dai and plant symptoms indicates that a screening of resistant genotypes cannot be performed at early infection times (e.g., 35 dai). For instance, some genotypes behaved as resistant up to at least 85 dai, after which they suffered an abrupt increase in symptoms and *V. dahliae* DNA content, which determined them to be finally classified as extremely susceptible (Figure 1A). This indicates that the plants can prevent proliferation of the fungus for a time, after which the barriers of resistance are overcome, and the disease eventually develops. This is in line with results showing that disease symptoms can appear long after planting olive cultivars in naturally infected soils (Trapero et al., 2013; Valverde et al., 2021). To postulate resistant genotypes as useful for the control of Verticillium wilt, it is necessary to assess the resistance of susceptible cultivars grafted on the resistant wild genotypes identified in this work. In this regard, we are currently evaluating the resistance of commercial cultivars grafted on different wild olive genotypes that have demonstrated resistance to the disease. Field evaluation in naturally infected soils is also needed to test the long-term persistence of the resistance trait.

In conclusion, we have identified 15 wild genotypes displaying similar or better resistance to Verticillium wilt than the resistant cultivar Frantoio. Measurement of Verticillium DNA content at early and late stages of infection, together with correlations with plant symptoms, made it possible to identify specific patterns of response in wild olive genotypes, pointing to the occurrence of different strategies of resistance to Verticillium wilt, such as avoidance and tolerance mechanisms. Therefore, this work represents a valuable source of resistant genotypes to be used as rootstocks and in breeding programs. Our findings contribute to the improvement of an integrated, effective, and sustainable strategy for optimal control of Verticillium wilt in the olive grove.

## Data Availability Statement

The raw data supporting the conclusions of this article will be made available by the authors when required, without undue reservation.

## Author Contributions

PD-R participated in all experimental tasks, particularly in plant material production, DNA quantification by qPCR and data analysis, as well as in writing the manuscript. AA contributed in the inoculation of the plants and participated in data analysis. LR-C contributed in the inoculation of the plants and DNA quantification by qPCR. NC designed the experiments, participated in inoculation of the plants, and wrote the manuscript. JC-F conceived the project, obtained the funds for its financing, and supervised the experiments and the manuscript. All authors contributed to the article and approved the submitted version.

## Conflict of Interest

The authors declare that the research was conducted in the absence of any commercial or financial relationships that could be construed as a potential conflict of interest.
