# Supplementary material for: Wild Olive Genotypes as a Valuable Source of Resistance to Defoliating Verticillium dahliae
Source: Front Plant Sci. 2021 Jul 1;12:662060. doi: 10.3389/fpls.2021.662060 (PMC8281240; doi:10.3389/fpls.2021.662060)
Supplement: Supplementary file 1 [file Data_Sheet_1.docx]

**Supplementary Files:**

**Supplementary Figure S1.** Progress of the severity of symptoms recorded in wild olive genotypes inoculated with the defoliating isolate VD117 of *Verticillium dahliae* (sum of assays 1 to 4). Values are the means of 24 plants per assay. Severity of symptoms was assessed each time on a 0-4 rating scale according to the percentage of *M*aximum *I*ntensity *S*ymptoms (*MIS*): chlorosis, leaf and shoot necrosis or defoliation: 0 = 0% *MIS* or no symptoms; 1 = 25% *MIS*; 2 = 50% *MIS*; 3 = 75% *MIS*; 4 = 100% *MIS* or dead plants. Reference control cultivars Picual, Arbequina and Frantoio are indicated as dashed lines.

**Supplementary Figure S2.** Evolution of morphological parameters with time in wild olive genotypes inoculated with *V. dahliae* isolate VD117 (A, C, E) or non-inoculated (B, D, F). Representative cultivars were also included: resistant Frantoio, moderately susceptible Arbequina and extremely susceptible Picual. Values of morphological parameters were normalized with the corresponding value at 0 dai. Results represent the average of the relative values measured in 12 plants per genotype and bars correspond to the standard error.

**Supplementary Figure S3.** Scatter plots including regression line, regression equation and coefficient of determination (R^2^) for the relationships between the Mean Normalized Quantity (MNQ) of *V. dahliae* DNA and the Final Mean Severity (FMS) (left graphics) or the RAUDPC (right graphics) at 120 dai. Results are the mean of 12 plants per assay, or 24 plants for those genotypes repeated in two different assays. Error bars in two dimensions indicate the standard error of the mean.

**Supplementary Figure S4. (A)** Scatter plots including regression line, regression equation and coefficient of determination (R^2^) for the relationships between the Mean Normalized Quantity (MNQ) of *Verticillium dahliae* DNA and the Final Mean Severity (FMS) (left graphic) or the RAUDPC (right graphic) at 120 days after inoculation (dai) in the group of resistant genotypes from assays 1, 2, 3 and 4. **(B)** Correlations between the Mean Normalized Quantity (MNQ) of *V. dahliae* DNA at 35 dai and the Final Mean Severity (FMS) at both 35 (left graphic) and 12 dai (right graphic) in the whole set of genotypes from assays 1, 2 and 3. Results are the mean of 12 plants per assay, or 24 plants for those genotypes repeated in two different assays. Error bars in two dimensions indicate the standard error of the mean.

**Supplementary Figure S5.** Quantification of *Verticillium dahliae* DNA (Mean Normalized Quantity, MNQ) in wild olive genotypes inoculated with the defoliating isolate VD117 of *Verticillium dahliae* at 35 and 120 days after inoculation (dai) in assays 1, 2 and 3. Bars represent the average values of 12-24 plants per genotype. Error bars indicate the standard error of the mean. The red line represents the average final mean severity (FMS) at 120 dai for each genotype.
